# *CTCFL* (*BORIS*) mRNA Expression in a Peripheral Giant Cell Granuloma of the Oral Cavity

**DOI:** 10.1155/2014/792615

**Published:** 2014-07-10

**Authors:** Graciela Zambrano-Galván, Miguel Reyes-Romero, Ronell Bologna-Molina, Oscar Eduardo Almeda-Ojeda, Obed Lemus-Rojero

**Affiliations:** ^1^Laboratory of Molecular Stomatology, Faculty of Dentistry, Universidad Juárez de Estado de Durango, Predio Canoas Street, 34067 Durango, Mexico; ^2^Department of Molecular Medicine, Faculty of Medicine and Nutrition, Universidad Juárez de Estado del Durango, Universidad Avenue, 34000 Durango, Mexico; ^3^Laboratory of Pathology and Bucal Medicine, Faculty of Dentistry, Universidad Juárez del Estado de Durango, Predio Canoas Street, 34067 Durango, Mexico; ^4^Faculty of Dentistry, Universidad Autónoma de Zacatecas, Begonias Street, Guadalupe, 98600 Zacatecas, Mexico

## Abstract

Peripheral giant cell granuloma (PGCG) is a relatively common benign reactive lesion of the oral cavity which can occur at any age. *CTCFL/BORIS* (*CTCF* like/Brother of the Regulator of Imprinted Sites) and *CTCF* (CCCTC-binding factor) are paralogous genes with an important role in the regulation of gene expression, genomic imprinting, and nuclear chromatin insulators regulation. *BORIS* expression promotes cell immortalization and growth while *CTCF* has tumor suppressor activity; the expression pattern may reflect the reverse transcription silencing of *BORIS*. The aim of this work was to describe a histopathological and molecular approach of an 8-year-old pediatric male patient with PGCG diagnosis. It was observed that the PGCG under study expressed *CTCF* as well as *BORIS* mRNAs alongside with the housekeeping gene *GAPDH*, which may be related to possible genetic and epigenetic changes in normal cells of oral cavity.

## 1. Introduction

Peripheral giant cell granuloma (PGCG) is a relatively common benign reactive lesion of the oral cavity which can occur at any age; it consists of a soft tissue injury mainly originating from the periosteum or periodontal membrane following local irritation or chronic trauma. The main clinical feature of PGCG is a red-purple nodule located in the region of the gums or the alveolar edentulous, mainly in the lower jaw [1, 2].

PGCC is of osteoclastic nature and is not considered a true neoplasm. It has been termed as PGCG “abnormal reparative”; however, this function has not been fully established, and its osteoclastic activity seems doubtful [3]. Although the presence of calcitonin membrane receptors, as well as osteoclastic activity, has been demonstrated by immunohistochemistry [4, 5], some authors report that the lesion is formed by cells of the fagocito mononuclear system [6]. In this respect, some authors propose that PGCG is a process in which the fibroblasts overexpress cytokines and growth factors, which induce or activate macrophages to become giant cells [7].

*CTCF* (CCCTC-binding factor) is an essential protein encoded by the gene of the same name, which is ubiquitously expressed and plays an important role in the regulation of gene expression; the multiple activities of* CTCF* in mammalians include transcriptional activation and repression, gene silencing, constitutive chromatin insulation, and functional reading of imprinted states, reasons for which it has been named the master weaver of the genome [8].* CTCF* has been proposed as a novel tumor suppressor gene because the* CTCF* expression suppresses tumor cell proliferation [9]. Some mutations in the* CTCF* gene have been localized and characterized in various types of cancer, including breast [10].

Recently was identified a paralogue of the* CTCF* gene that was called* CTCFL* (*CTCF* like) and also* BORIS* (Brother of the Regulator of Imprinted Sites, name that will be used in this paper), which encodes a protein of the same name [11].* CTCF* and* BORIS* are zinc-finger proteins sharing the same 11 zinc fingers and interact with similar DNA sequences, although they have different amino and carboxyl ends. Normally* CTCF* and* BORIS* are expressed in a mutually exclusive pattern that correlates with the reestablishment of methylation marks during male germ cell differentiation [11, 12].

Apart of male germ cells, scarce or null expression of* BORIS* has been reported in normal human tissues and cells. Thus, it has been suggested that* BORIS *expression is controlled at epigenetic level by promoter DNA methylation, and their activation requires demethylation [13]. In cancer patients, high expression of* BORIS* is correlated with the size and grade of the tumor, especially in breast, endometrium, prostate, and colon [14]. An inhibitor of DNA methylation, 5-aza-2′deoxy-cytidine (5-azadC), as well as histone deacetylase inhibitors, induces or enhances the* BORIS* gene expression in various carcinoma cell lines [15].

## 2. Case Report

An 8-year-old pediatric male patient, referred from private practice to the Department of Clinical Pathology of the Faculty of Dentistry of the Juarez University of the State of Durango, Mexico, presented a lobular nodular lesion at the level of dental organs 31 and 32, similar in color to the mucosa, with a pediculated base in the gingival region, with an evolution time of approximately 6 months. The patient underwent excisional biopsy; the specimen was grayish-white, irregularly shaped, and had a firm consistency. The measures of the specimen were 1.1 × 0.6 × 0.7 cm. The sample was formalin-fixed and paraffin-embedded (FFPE) for histopathological and molecular analysis.

**Figure 1:**
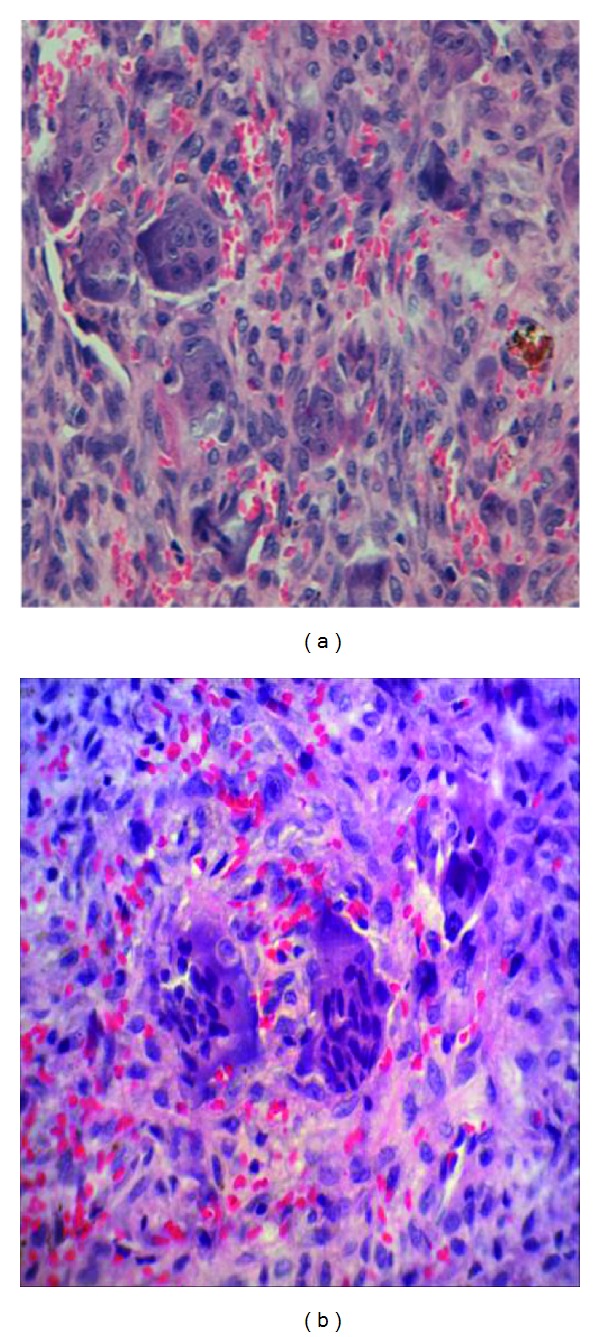
Two photomicrography images of the peripheric giant cell granuloma showing numerous giant cells with mitotic nuclei (H&E staining, ×65).

*Microscopic Description*. H&E staining was performed and analyzed with a Leica DMD108 Optic Microscopic. By H&E staining a stratified squamous parakeratinized epithelium, with the presence of elongated epithelial anastomosing nails and pseudoepitheliomatous hyperplasia, was observed. Epithelial atrophic areas presenting a continuum with the stroma, dense fibrous hyalinized connective tissue, heavy hemorrhagic areas, blood vessels, and the presence of multinucleated giant cells, some of which with vesicular nuclei and abundant mitotic figures, were also observed. Furthermore, a lymphoplasmocitary inflammatory response dominated by positive surgical margins was present (Figure 1).

*Molecular Description*. A descriptive study of a FFPE sample with histopathologic diagnosis of PGCG was conducted. Total RNA was obtained with the QuickExtract FFPE RNA Extraction Kit (Illumina, San Diego, CA) and cDNA was synthesized with the cDNA Synthesis Kit iScript (BIO-RAD).* BORIS*,* CTCF, *and* GAPDH *(used as housekeeping gene) mRNA expression was carried out by real-time PCR using the QuantiTect SYBR Green PCR kit system and Quantitect Primers (Qiagen Germantown, MD. Catalogue numbers:* BORIS*, QT00023191, amplicon size 125 bp;* CTCF*, QT00045437, amplicon size 119 bp;* GAPDH*, QT01192646, amplicon size 119 bp).

The amplification conditions consisted of incubation at 50°C for 30 min, followed by polymerase activation at 95°C for 15 min and 45 cycles of denaturation at 94°C for 15 s, and annealing and extension at 60°C for 1 min. Amplification, acquisition, and data analysis were performed with an Eco Illumina Real-Time PCR System and Eco Version 0.17.53.9 Software.

The specificity of the reaction was verified by melting analysis of the amplified products; the size of the amplicons was analyzed by electrophoresis in agarose gels stained with ethidium bromide, because we use a SYBR Green as intercalating dye chemistry instead of allele-specific probes.

**Figure 2:**
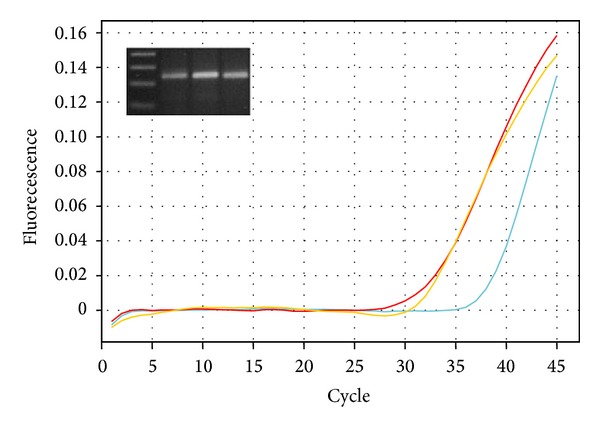
Amplification curves for* BORIS, CTCF, *and* GAPDH *genes in blue, yellow, and red, respectively, as obtained with Quantitect (Qiagen) primers. The insert shows the PCR products resolved by gel electrophoresis; from left to right, 50 bp ladder,* BORIS *(125 bp),* CTCF *(119 bp), and* GAPDH *(119 bp).

It was observed that the peripheral giant cell granuloma under study expressed* CTCF* as well as* BORIS* mRNAs alongside with the housekeeping gene* GAPDH*. The cycle threshold (Ct) value in the linear part of the amplification was 31.14 for* GAPDH*, 32.06 for* CTCF,* and 35.34 for* BORIS*; this means that* CTCF* had 0.52 and* BORIS* had 0.05 times the expression value of* GAPDH* used as housekeeping gene. Thus, the* CTCF/BORIS* expression ratio was 10.4. The amplification kinetics and the end products of real-time PCR are presented in Figure 2.

## 3. Discussion

*BORIS* expression has been reported in various types of carcinoma such as breast, ovary, colon, and prostate, but to date there are no data regarding its expression in benign or malignant oral cancer.

PGCG is a benign lesion of the oral cavity which has a recurrent behavior. There are no data regarding* CTCF* and* BORIS* expression in this kind of lesion. In this work, it was found that in a case of PGCG there existed coexpression at transcriptional level of both genes. Although the role of* BORIS* in cancer has not been elucidated to date, divergent roles have been proposed. On one hand, it is believed that* BORIS* could participate in the molecular mechanisms driving cancer behaving as oncogene [16]; on the other hand, an ancillary role when* CTCF* is mutated and the encoded protein is dysfunctional has been proposed in a recent report [9]. PGCG is a benign lesion in which both scenarios are possible, but deeper genetic and epigenetic studies are necessary to shed light on this matter.

This is the first report showing* BORIS *expression in a benign lesion of the oral cavity and, as a starting point, the issue merits further studies.

## 4. Conclusion

The coexpression of* BORIS* and* CTCF* genes in PGCG may be associated with the alteration of genetic and epigenetic mechanisms in the normal function of the cells in the oral cavity.

## References

[1] Chaparro-Avendaño AV, Berini-Aytés L, Gay-Escoda C (2005). Peripheral giant cell granuloma. A report of five cases and review of the literature.. *Medicina Oral, Patologia Oral y Cirugia Bucal*.

[2] Adlakha VK, Chandna P, Rehani U, Rana V, Malik P (2010). Peripheral giant cell granuloma. *Journal of Indian Society of Pedodontics and Preventive Dentistry*.

[3] Bonetti F, Pelosi G, Martignoni G (1990). Peripheral giant cell granuloma: evidence for osteoclastic differentiation. *Oral Surgery Oral Medicine and Oral Pathology*.

[4] Lim L, Gibbins JR (1995). Immunohistochemical and ultrastructural evidence of a modified microvasculature in the giant cell granuloma of the jaws. *Oral Surgery, Oral Medicine, Oral Pathology, Oral Radiology and*.

[5] Carvalho YR, Loyola AM, Gomez RS, Araújo VC (1995). Peripheral giant cell granuloma. An immunohistochemical and ultrastructural study.. *Oral Diseases*.

[6] Tandon PN, Gupta SK, Gupta DS, Jurel SK, Saraswat A (2012). Peripheral giant cell granuloma. *Contemporary Clinical Dentistry*.

[7] Gándara JM, Pacheco JL, Gándara P (2002). Granuloma periférico de células gigantes. Revisión de 13 casos clínicos. *Medicina Oral*.

[8] Phillips JE, Corces VG (2009). CTCF: master weaver of the genome. *Cell*.

[9] Rasko JEJ, Klenova EM, Leon J (2001). Cell growth inhibition by the multifunctional multivalent zinc-finger factor CTCF. *Cancer Research*.

[10] Filippova GN, Qi CF, Ulmer JE (2002). Tumor-associated zinc finger mutations in the CTCF transcription factor selectively alter its DNA-binding specificity. *Cancer Research*.

[11] Loukinov DI, Pugacheva E, Vatolin S (2002). BORIS, a novel male germ-line-specific protein associated with epigenetic reprogramming events, shares the same 11-zinc-finger domain with CTCF, the insulator protein involved in reading imprinting marks in the soma. *Proceedings of the National Academy of Sciences of the United States of America*.

[12] Klenova EM, Morse 3rd. HC, Ohlsson R, Lobanenkov VV (2002). The novel BORIS+CTCF gene family is uniquely involved in the epigenetics of normal biology and cancer. *Seminars in Cancer Biology*.

[13] Link PA, Zhang W, Odunsi K, Karpf AR (2013). BORIS/CTCFL mRNA isoform expression and epigenetic regulation in epithelial ovarian cancer. *Cancer Immunity*.

[14] Martin-Kleiner I (2012). BORIS in human cancers: a review. *European Journal of Cancer*.

[15] Tiffen JC, Bailey CG, Marshall AD (2013). The cancer-testis antigen BORIS phenocopies the tumor suppressor CTCF in normal and neoplastic cells. *International Journal of Cancer*.

[16] de Necochea-Campion R, Ghochikyan A, Josephs SF (2011). Expression of the Epigenetic factor BORIS (CTCFL) in the Human Genome. *Journal of Translational Medicine*.

